# Management of Total Transection of Nasoendotracheal Tube during LeFort I Osteotomy

**DOI:** 10.1155/2020/2097240

**Published:** 2020-11-17

**Authors:** Miles Somers, Peter Tsakiris, Peter Isert, Samuel Kim

**Affiliations:** ^1^Department of Oral and Maxillofacial Surgery, Prince of Wales Hospital, Randwick, NSW, Australia; ^2^VMO Anaesthetist, Prince of Wales Private Hospital, Randwick, NSW, Australia

## Abstract

Transection of the nasoendotracheal tube during orthognathic surgery is a rare, but life-threatening complication. We present a case of complete nasoendotracheal tube transection during a LeFort 1 osteotomy and discuss appropriate preventative and management techniques.

## 1. Introduction

Complete transection of the nasoendotracheal tube (NET) during surgery is rare [1]. However, it poses a potentially life-threatening complication that must be managed effectively. There are several reported cases in the literature reporting both complete [2, 3] and partial transection [4–7]. Maxillofacial surgery, in particular orthognathic surgery, is carried out in close proximity to endotracheal tubes and therefore may be prone to damage during surgery. The previously reported cases have occurred when operating on different areas, such as the palate [2], lateral nasal wall [3], and maxilla [4, 5], as well as immediately following the pterygomaxillary dysjunction [8]. Different instruments have been implicated in these complications, such as an osteotome [3, 8], drill [6], harmonic scalpel [9], Gigli saw [2], and a pneumatic saw [4, 5]. Both the surgeon and anaesthetist must be aware of the possible complication, requiring teamwork and communication between surgeon and anaesthetist in order to manage this complication. In this paper, we discuss a case of complete transection of a NET during a 2-piece LeFort I osteotomy and suggest preventative measures and management of this complication.

## 2. Case Report

A 28-year-old male underwent a 2-piece maxillary osteotomy plus right ramus graft for genioplasty. The aim of the surgery was orthognathic correction of a dentoskeletal class III malocclusion related to hypoplasia of the maxilla secondary to cleft lip and palate. The patient had a previous cleft repair. The medical history was otherwise unremarkable.

**Figure 1:**
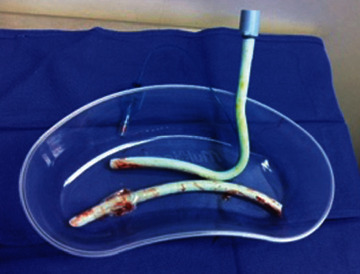
Severed nasoendotracheal tube.

**Figure 2:**
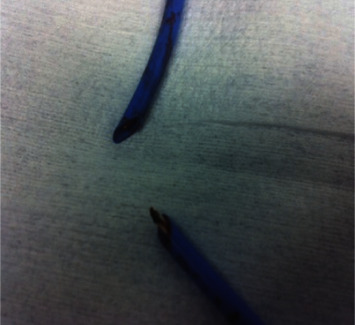
Severed nasal temperature probe.

The patient was anaesthetised, paralysed, and easily intubated with a size 6.5 soft, north-facing Nasal RAE NET (Portex PolarTM Preformed Tracheal Tube). A ribbon gauze throat pack and a nasal temperature probe were then placed by the anaesthetist. An incision was made from tooth 16 to tooth 26, and a full thickness mucoperiosteal flap was raised to skeletonise the nasal floor, inferior septum, and lateral nasal walls. Bone cuts were made with a piezotome, and the maxilla was cut between teeth 12 and 13 for a 2-piece LeFort I osteotomy. Prior to maxillary downfracture, the pterygomaxillary junction and nasal septum were split with an osteotome. At the time of surgery, however, the usual thin-guarded septal osteotome was unavailable, and hence a larger ear, nose, and throat septal osteotome without elongated protective lateral stops was used. A finger was placed through the mouth for detecting and stopping the osteotome in the posterior nasal pharynx as per usual practice. However, in this case, the abnormal and thickened soft tissue from previous pharyngoplasty and cleft meant that the assistant was not able to place the finger sufficiently in the posterior nasal cavity to detect the osteotome. Soon after the split of the nasal septum, the anaesthetist noted a sudden loss of monitored capnography associated with emptying of the ventilator bellows. The anaesthetist immediately commenced manual bag ventilation through the NET and noted a huge air leak from the tube and an inability to raise the chest with bag ventilation. At this point, bag-tube ventilation of the chest was minimal but improved when the surgeon closed the mouth and prevented this air leak from escaping. Only mild desaturation to 88% occurred. Emergency nasal extubation then revealed a completely severed NET (Figure 1) and nasal temperature probe (Figure 2), and at direct laryngoscopy via Macintosh blade, the distal segment of the endotracheal tube was then removed with a Magills forceps. The throat pack remained in situ as the patient was promptly reintubated through the same nostril. A nasal temperature probe was also reinserted. The oxygen saturation and capnograhy trace then promptly returned to normal. The remainder of the procedure was then carried out without further complication, and the patient was discharged 3 days postoperatively. At the end of the procedure, the posterior pharynx was inspected both directly under vision and then indirectly using a video laryngoscope. This revealed a 1 cm blunt laceration on the posterior pharyngeal wall that healed uneventfully.

## 3. Discussion

By using our own experience of complete transection, in addition to other reported cases in the literature, we suggest a number of factors to help prevent such complications during orthognathic surgery.

Firstly, the common factor in previously reported cases was the use of sharp instruments to cut bone, such as pneumatic saws, drills, and Gigli saws. Therefore, we propose the use of peizotome as a safer instrument as it is unable to cut through the endotracheal tube as opposed to conventional saws. Although, in this reported case, the tube was not transected by a saw, we believe it to be a good preventative measure in avoiding this complication when performing the lateral nasal split.

The use of a soft NET potentially poses a higher risk of transection compared to a reinforced NET with internal wiring. The soft NETs are generally preferred as they have the advantage of being able to be placed north-facing and allow better intraoperative access.

3-Dimensional scans should be examined thoroughly preoperatively for appropriate surgical planning and visualisation of the proximity of cuts to the anticipated location of the NET. For example, in our case, the fibrosis from the surgical repair of the pre-existing cleft deformity tended to guide the tube more centrally posterior to the nasal septum, hence being in the direct line of the osteotome during the nasal septal split. Furthermore, due to the previous cleft surgery, the soft tissue of the palate was fibrosed. This made it difficult to palpate the tip of the advancing osteotome through this thickened soft tissue. Interestingly, the case report by Bigdoli et al. was also on a cleft patient suggesting that cleft patients may present a higher risk for this type of complication due to altered anatomy. It has also been suggested that the anatomy of maxillary excess or prognathism creates challenging airway management and may contribute to increased risk of endotracheal damage intraoperatively [6].

**Figure 3:**
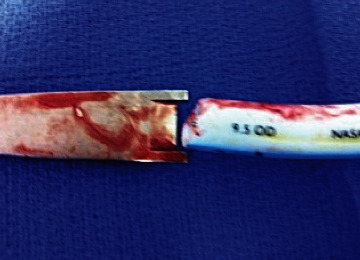
Relationship of size of osteotome to nasoendotracheal tube.

This case is the first reported case of a NET transection occurring during a nasal septal split. Hosseini Bidgoli et al. described a transection during lateral nasal wall split [3]. The common theme between this case and ours was the use of an unguarded osteotome. The use of a guarded osteotome with lateral stops that is thinner than the NET is perhaps one of the most crucial measures to avoid this complication. As can be seen in Figure 3, the osteotome was of a similar diameter to the tube. We, therefore, highlight the importance of appropriately selecting osteotomes prior to surgery to eliminate the risk of transection caused during the osteotomy. Other authors have also modified their technique of a maxillary osteotomy to eliminate the use of an osteotome for the septal split in order to avoid this complication [10].

If this complication does occur, it must be managed immediately to prevent a potentially devastating outcome. Immediate recognition and avoidance of delay in re-establishing a protected airway requires coordinated efforts between the surgeon and anaesthetist. In our case, reintubation was performed uneventfully due to a clear operative site, good suction of the airway, and favourable anatomy. However, Ladi and Aphale managed their complete transection via an emergency tracheostomy because they anticipated that removing the distorted tube with intact nylon rings would lead to hypoxia, aspiration, and airway trauma [2]. Similarly, difficulties of reintubation due to poor visibility, bleeding, edges of cut tube catching on bone, and poorly defined tissue planes have been suggested [3]. Fortunately, in our case, we were able to reintubate the patient, whilst other cases have reintubated using a tube changer [6] or a gum elastic bougie [4]. An interim attempt to seal the mouth and in so reducing air leak during ventilation might allow gas to flow between the two cut ends of the tube and provide at least some ventilation. Curiously, this may be most beneficial as in our case if the throat pack is left in as it occludes the oesophagus rather than the larynx, thereby allowing anaesthetic gases to pass through the “path of least resistance” and provide at least some interim ventilation and oxygenation until emergency reintubation is attempted. This technique must be terminated, however, if there is no adequate ventilation of the chest or if obvious gastric inflation occurs during this attempt. In a similar reported case, the anaesthetist chose to pack the larynx and leave the damaged NET in situ for the remainder of the case (ten minutes) as ventilatory parameters and saturations were still normal [7]. The surgeon should aim to clear the surgical field adequately to allow good visualisation so that reintubation can be attempted. In the event that reintubation is deemed not possible or too difficult, a surgical airway should be considered [2, 6].

## 4. Conclusion

Immediate recognition and coordinated efforts from the surgeon and anaesthetist when dealing with this rare complication resulted in no adverse outcome for the patient. Equipment management, a heightened sense of awareness, cessation of all other activities and restoration of a patent airway are of primary importance. We suggest that careful preoperative planning and the use of piezotomes and narrow, guarded osteotomes when performing LeFort osteotomies must be strictly adhered to. In addition, a trial of bag-tube ventilation through the open ends of the transected NET with the mouth sealed may be a useful interim procedure if this complication occurs.
